# The Cortical Asymmetry Index for subtyping dementia patients

**DOI:** 10.1007/s00330-025-11400-y

**Published:** 2025-02-11

**Authors:** Agnès Pérez-Millan, Uma Maria Lal-Trehan Estrada, Neus Falgàs, Núria Guillén, Sergi Borrego-Écija, Jordi Juncà-Parella, Beatriz Bosch, Adrià Tort-Merino, Jordi Sarto, Josep Maria Augé, Anna Antonell, Núria Bargalló, Raquel Ruiz-García, Laura Naranjo, Mircea Balasa, Albert Lladó, Roser Sala-Llonch, Raquel Sánchez-Valle

**Affiliations:** 1 Alzheimer’s Disease and Other Cognitive Disorders Group, Service of Neurology, Hospital Clínic de Barcelona, Fundació Recerca Clínic Barcelona-IDIBAPS, 08036 Barcelona, Spain; 2 Institut de Neurociències, University of Barcelona, 08036 Barcelona, Spain; 3 Department of Biomedicine, University of Barcelona, 08036 Barcelona, Spain; 4 Biochemistry and Molecular Genetics Department, Hospital Clínic de Barcelona, 08036 Barcelona, Spain; 5 Image Diagnostic Centre, Hospital Clínic de Barcelona, Barcelona, Spain; 6 CIBER de Salud Mental, Instituto de Salud Carlos III, Magnetic Resonance Image Core Facility, IDIBAPS, 08036 Barcelona, Spain; 7 Immunology Service, Biomedical Diagnostic Center, Hospital Clínic de Barcelona, 08036 Barcelona, Spain; 8 Centro de Investigación Biomédica en Red de Bioingeniería, Biomateriales y Nanomedicina (CIBER-BBN), Barcelona, Spain; 9 Institute of Biomedical Research August Pi i Sunyer (IDIBAPS), 08036 Barcelona, Spain; 10 Departament de Medicina, Facultat de Medicina i Ciències de la Salut, Universitat de Barcelona, 08036 Barcelona, Spain

**Keywords:** Brain, Alzheimer’s disease, Frontotemporal dementia, Magnetic resonance imaging

## Abstract

**Objectives:**

Frontotemporal dementia (FTD) usually shows more asymmetric atrophy patterns than Alzheimer’s disease (AD). We aim to quantify this asymmetry to differentiate FTD, AD, and FTD subtypes.

**Methods:**

We studied T1-MRI scans, including FTD (different phenotypes), AD, and healthy controls (CTR). We defined the Cortical Asymmetry Index (CAI) using measures based on a metric derived from information theory with the cortical thickness measures. Some participants had additional follow-up MRIs, cerebrospinal fluid (CSF), or plasma measures. We analysed differences at cross-sectional and longitudinal levels. We then clustered FTD and AD participants based on the CAI values and studied the patients’ fluid biomarker characteristics within each cluster.

**Results:**

A total of 101 FTD patients (64  ±  8 years, 53 men), 230 AD patients (65  ±  10 years, 84 men), and 173 CTR (59  ±  15 years, 67 men) were studied. CAI differentiated FTD, AD, and CTR. It also distinguished the semantic variant primary progressive aphasia (svPPA) from the other FTD phenotypes. In FTD, the CAI increased over time. The cluster analysis identified two subgroups within FTD, characterised by different neurofilament-light (NfL) levels, and two subgroups within AD, with different plasma glial fibrillary acidic protein (GFAP) levels. In AD, CAI correlated with GFAP and Mini-Mental State Examination (MMSE); in FTD, the CAI was associated with NfL levels.

**Conclusions:**

The proposed method quantifies asymmetries previously described visually. The CAI could define clinically and biologically meaningful disease subgroups in the differential diagnosis of AD and FTD and its subtypes. CAI could also be of interest in tracking disease progression in FTD.

**Key Points:**

***Question*** *There is a need to find quantitative metrics from MRI that can identify disease subgroups, and that could be useful for diagnosis and tracking.*

***Findings*** *We propose a Cortical Asymmetry Index that differentiates Alzheimer’s disease (AD) from Frontotemporal dementia (FTD), distinguishes FTD subtypes, correlates with NFL and GFAP levels, and monitors FTD progression.*

***Clinical relevance*** *Our proposed index holds the potential to support clinical applications for diagnosis and disease tracking in AD and FTD, using a quantitative summary metric from MRI data. It also contributes to the understanding of these diseases.*

## Introduction

Frontotemporal dementia (FTD) is a clinically, pathologically, and genetically heterogeneous neurodegenerative disorder associated with frontal and temporal atrophy. FTD patients tend to be misdiagnosed with Alzheimer’s disease (AD), especially at relatively young ages, although early behavioural and personality changes typical of FTD are not common in initial AD [1–3]. However, AD is the most frequent dementia and sometimes is the first choice for many non-specialist clinicians. For this reason, there is a need to identify tools to help accurately diagnose dementia’s underlying etiologies and their subtypes. Cortical asymmetry has been associated with characteristically clinical features in psychiatric and neurological conditions [4, 5]. In this sense, FTD patients show a more asymmetrical pattern at visual inspection compared to AD [6–9].

Within the FTD phenotypes, the semantic variant of primary progressive aphasia (svPPA) is the most asymmetric [6, 10, 11]. In this sense, the study of brain asymmetry could help in the diagnosis of the different phenotypes of FTD: the behavioural variant frontotemporal dementia (bvFTD) or the FTD-related primary progressive aphasia phenotypes, including svPPA and nonfluent variant primary progressive aphasia (nfvPPA) [12, 13]. A measure of cortical asymmetry could help in the early differential diagnosis or monitoring of the progress of neurodegeneration [5, 14].

The differences between structural measures from the left and right hemispheres can be detected in structural magnetic resonance imaging (MRI) [4, 5, 15]. There is no established way to calculate the asymmetry, and different approaches have been used in multiple works on various brain diseases [14, 16–21]. Usually, these approaches use mean values across hemispheres or measures derived from linear arithmetic. Then, computational algorithms can obtain measures of cortical asymmetry from the global and regional cortical thickness (CTh), surface area, or subcortical volumes. Previously published works indicate that approaches using the probability distribution to obtain an asymmetric index could get better results and are currently being used in other biological areas [22–24]. Using a probability distribution-based asymmetric index to evaluate cortical asymmetry offers advantages in terms of robustness to variability, statistical power, capturing the probabilistic nature of the data, handling distributional changes, and robustness to outliers. However, it also presents challenges related to computational complexity, higher data requirements, and potential interpretation challenges.

In this study, we first defined the Cortical Asymmetry Index (CAI) using measures derived from the information theory with CTh measures. Secondly, we aimed to study if CAI might be used to differentiate FTD and AD patients and the different FTD clinical expressions. Thirdly, we wanted to study cortical asymmetry changes at the longitudinal level. Finally, we explored the capability of the CAI to identify subgroups and its correlation with fluid biomarkers and cognitive measures. All these analyses were done with the objective of evaluating brain asymmetry as a key tool to help in the differential diagnosis between dementias or subtypes and to track the progression of different types of dementia.

## Materials and methods

### Study population

The prospective study includes 554 participants recruited from the AD and other cognitive disorders group of the Hospital Clínic de Barcelona (HCB), Barcelona, Spain. This study was performed according to the international consensus for research with human subjects (the updated version of Helsinki’s Statement, Fortaleza, 2013) and Spanish regulations. The HCB Ethics Committee approved the study (HCB 2019/0105), and all the participants signed the informed consent. All participants underwent a complete clinical and cognitive evaluation and a 3 T high-resolution structural MRI scan. A subset of the study participants had available follow-up visits, cerebrospinal fluid (CSF) or plasma measures, and Mini-Mental State Examination (MMSE).

We included 173 healthy controls (CTR) (96 with follow-up MRI scans with two years approximately between scans), 230 AD (29 with follow-up MRI scans with two years approximately between scans), including amnesic and non-amnesic participants, and 101 FTD (29 with follow-up MRI scans with one year and a half approximately between scans). FTD participants were distributed as follows: 55 bvFTD (14 follow-up MRI scans), 21 nfvPPA (7 follow-up MRI scans), and 24 svPPA (9 follow-up MRI scans).

All AD participants fulfilled the criteria for mild cognitive impairment due to AD or mild dementia due to AD [25, 26] supported by the AD CSF biomarkers profile (including amyloid-beta protein 42 (Aβ42), p-tau and t-tau levels), suggesting underlying AD neuropathology according to the National Institute on Aging/Alzheimer’s Association Research Framework 2018 [27]. The FTD participants fulfilled the diagnostic criteria for bvFTD, svPPA, or nfvPPA [12, 13]. The CTR included both individuals with and without subjective memory complaints. All of them performed within the normal range in all the tests of the comprehensive neuropsychological assessment. Participants with a history of stroke, traumatic brain injury, tumour, major psychiatric disorder, or alcohol abuse were excluded.

AD participants were 64% women, and the mean age was 65.3 ± 9.7; FTD participants were 48% women, the mean age was 63.7 ± 8.4, and CTR participants were 61% women, and the mean age was 59.4 ± 15.0. As expected, CSF and plasma biomarkers levels showed significant differences between groups, except for plasma t-tau and UCH-L1 (Table 1).

**Table 1:** Group summaries are written as each measure’s mean and standard deviation

	CTR	AD	FTD	CTR-AD *p*-values	CTR-FTD *p*-values	AD-FTD *p*-values
*N* MRI	173	230	101	---	---	---
Sex at MRI, Men/Women	67/106	84/146	53/48	---	---	---
Age at MRI, years (SD)	59.4 (15.0)	65.3 (9.9)	63.7 (8.3)	---	---	---
CSF NfL, pg/mL (SD)	536.1 (312.6)	1106.9 (570.4)	2340.6 (1736.3)	**1.6e-7**	**< 2e-16**	**< 2e-16**
CSF 14-3-3, pg/mL (SD)	2531.9 (748.2)	5727.3 (2303.5)	4234.9 (1869.1)	**< 2e-16**	**3.0e-6**	**5.9e-6**
CSF YKL-40, pg/mL (SD)	270.1 (123.0)	328.6 (132.9)	315.3 (127.0)	**0.0028**	0.15	0.62
Plasma t-tau, pg/mL (SD)	4.4 (4.4)	4.2 (1.7)	3.7 (1.3)	0.65	0.65	0.65
Plasma p-tau, pg/mL (SD)	2.4 (2.6)	5.4 (7.7)	1.3 (1.0)	**0.0011**	0.37	**0.0019**
Plasma NfL, pg/mL (SD)	9.2 (6.6)	15.0 (6.5)	21.2 (15.9)	**1.7e-6**	**2.2e-9**	**8.0e-4**
Plasma GFAP, pg/mL (SD)	96.5 (64.9)	252.6 (154.6)	154.6 (101.7)	**1.0e-15**	**0.041**	**0.00066**
Plasma UCH-L1, pg/mL (SD)	35.1 (76.7)	17.5 (17.6)	22.1 (14.4)	0.15	0.71	0.44

We calculated differences between groups with the ANOVA test for all the variables except sex and age. We highlighted the significant group differences in bold. We measured pairwise differences with a Benjamini–Hochberg. *p*-values shown are corrected for multiple comparisons

### Biochemical markers

We used commercially available single-analyte enzyme-linked immunosorbent assay (ELISA) kits to determine levels of CSF neurofilament-light chain (NfL) (IBL International) and CSF 14-3-3 (CircuLex, MBL International Corporation) at the AD and other cognitive disorders group laboratory, Barcelona, Spain.

Plasma biomarker concentrations were measured using single-molecule array (SIMOA), Quanterix Neurology 4-Plex A including total tau (t-tau), glial fibrillary acidic protein (GFAP), NfL, and Ubiquitin C-terminal hydrolase L1 (UCH-L1) and the Quanterix p-tau181 Advantage V2 and V2.1 assays following the manufacturer’s protocol (Quanterix), we harmonised the values of the two kits.

### MRI acquisition

We acquired a high-resolution 3D structural dataset (T1-weighted, MP-RAGE, repetition time = 2.300 ms, echo time = 2.98 ms, 240 slices, field-of-view = 256 mm, voxel size = 1 × 1 × 1 mm) for everyone at each time point in a 3 T Magnetom Trio Tim scanner (Siemens Medical Systems) upgraded to a 3-T Prisma scanner (Siemens Medical Systems) during the study.

### MRI processing

We used the processing stream available in FreeSurfer version 6.0 (http://surfer.nmr.mgh.harvard.edu.sire.ub.edu/) to perform cortical reconstruction and volumetric segmentation of the T1-weighted acquisitions. FreeSurfer allowed us to generate automated CTh maps. First, it obtained a surface deformation, which enables the detection of tissue boundaries, and then CTh is estimated as the distance between the white and pial surface. The FreeSurfer pipeline also performs automated parcellation of the cortex using available atlases. Details of the full pipeline are reported elsewhere [28, 29]. For this study, we used the mean CTh measures in 68 cortical parcellations (34 per hemisphere) derived from the Desikan-Killiany atlas available in FreeSurfer [30]. All images and individual segmentations were visually inspected and manually corrected if needed.

### Cortical Asymmetry Index (CAI)

We obtained the CAI by implementing a measure derived from the Jensen-Shannon distance (JSD), a methodology based on a metric derived from information theory [31]. Differently from the original JSD definition, we did not normalise the probability density function. The JSD measures the difference between two probability distributions (P1 and P2) using the square root of the Jensen-Shannon divergence, which is based on the Kullback-Leibler divergence (KLD). The KLD is also referred to as relative entropy between two distributions. It is calculated as the negative sum of the probability of each event in P1 multiplied by the logarithm of the probability of the same event in P2 divided by the probability of the event in P1. The JSD is the symmetric version of KLD, so the JSD from one probability P1 to another probability P2 is the same as the JSD of P2 from P1. We obtained the CAI using the distribution of CTh measures of each brain hemisphere (previously obtained with the software FreeSurfer) that represents the difference in CTh distribution between brain hemispheres. We used raw CTh values to calculate the CAI without any subject normalisation, as the index is calculated individually for each subject across hemispheres, which mitigates inter-subject variability. Thus, the CAI allows us to quantify brain structural asymmetry at the individual level. CAI is a non-dimensional measure with higher values indicating a more asymmetric brain.

The CAI was calculated using in-house methods implemented in Python version 3.10.6 (www.python.org). The code for estimating the CAI is available at https://github.com/Agnes2/CAI.

### Statistical analysis

We first compared the CAI between all groups, including CTR, AD, and FTD patients. Then, we performed comparisons across the different FTD phenotypes. In both cases, we used the ANOVA test, corrected by age, and a post hoc analysis with the Tukey test to correct for multiple comparisons. Finally, we performed longitudinal analyses for participants with two visits with linear mixed effects models (LME) for each clinical group to study changes between visits; we added age at MRI, sex, and age of onset (for AD and FTD) as fixed effects.

We then analysed the CAI using cluster analyses for FTD and AD participants separately to evaluate whether subgroups emerged within each disease. We used hierarchical clustering, precisely agglomerative nesting cluster with Ward’s method and Manhattan metric. We determined the optimal number of clusters in each case with the Silhouette strategy. Then, with the cluster groups obtained, we studied differences in fluid biomarkers across subgroups with non-parametric tests such as the Kruskal Wallis or Wilcoxon tests. All biomarkers’ levels were converted to *z*-score before these calculations.

Finally, we evaluated the association between CAI and the CSF and plasma biomarkers that emerged in the cluster analysis. In addition, we studied the correlation of CAI with age, and MMSE. We used Spearman’s rank correlation coefficient due to the limited samples.

We implemented the statistical and cluster analyses (with cluster and factoextra packages) in R version 4.2.1. The significance level was set in all the analyses at a *p*-value < 0.05.

## Results

### Cross-sectional CAI differences

**Fig. 1:**
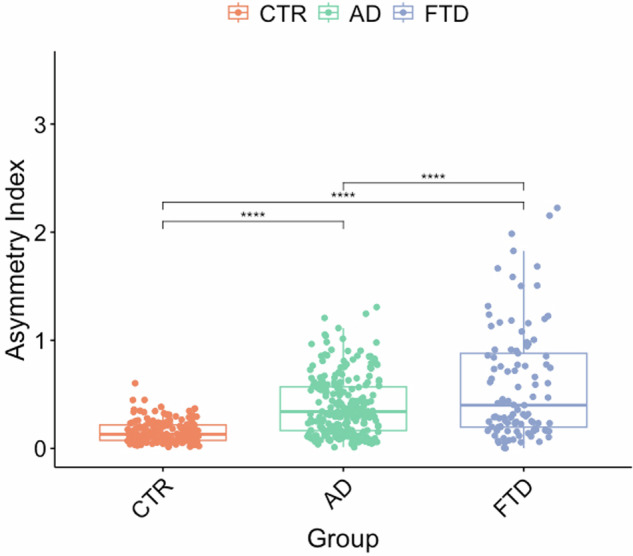
Asymmetric index significant differences between Alzheimer’s disease patients (AD) and frontotemporal dementia patients (FTD) patients and healthy controls (CTR). The symbol represents ****p < 0.0001

AD participants had a CAI of 0.40 ± 0.28, FTD participants 0.59 ± 0.52, and CTR 0.16 ± 0.10. We found higher CAI in AD and FTD patients than CTR (adjusted *p*-value < 0.0001), indicating a more asymmetric brain structure. FTD participants also presented a higher CAI than AD patients (adjusted *p*-value < 0.0001) (Fig. 1). The possible effects of sex, brain size, and mean CTh on CAI have been studied and are nonexistent (see Supplementary Material).

**Fig. 2:**
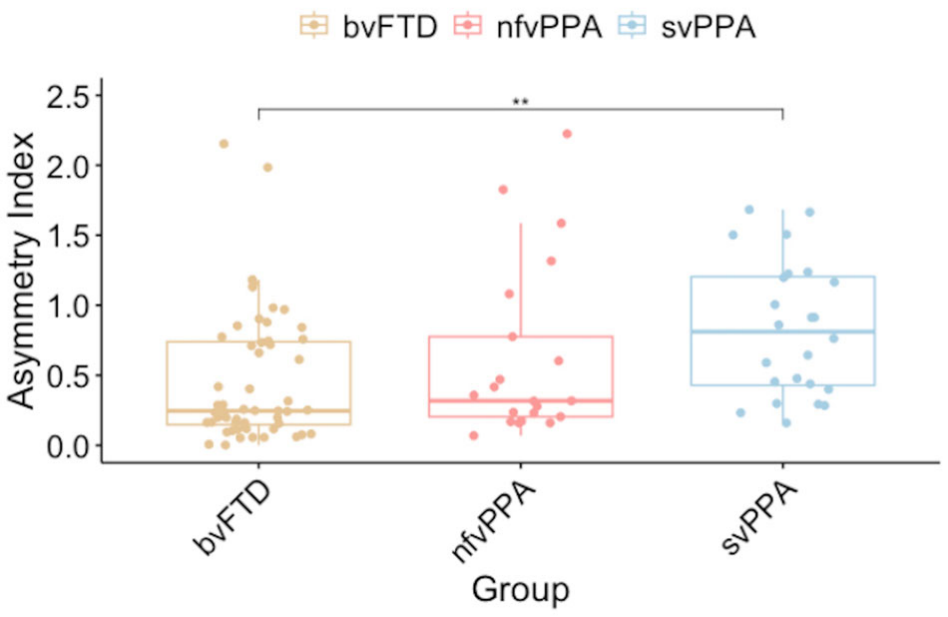
Asymmetric index significant differences frontotemporal dementia patients (FTD) variants. The symbol represents **p < 0.01

Then, we studied the CAI across FTD clinical phenotypes. The bvFTD patients had a mean CAI of 0.46 ± 0.46, nfvPPA patients 0.62 ± 0.62, and svPPA patients 0.83 ± 0.48. When studying the differences between FTD clinical phenotypes, svPPA presented higher CAI values than bvFTD (adjusted *p*-value < 0.01) (Fig. 2).

### Longitudinal changes in CAI

**Fig. 3:**
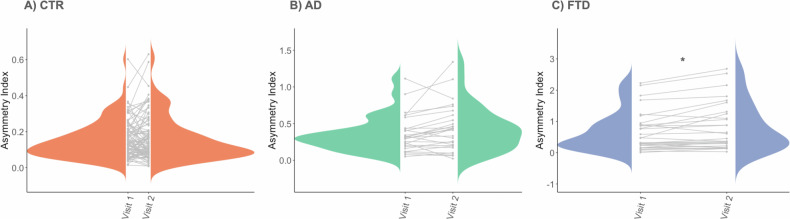
The spaghetti plot shows the individual trajectories between visit 1 and visit 2. It includes the density histogram for each visit. The symbol represents *p < 0.05. **A** Healthy controls (CTR), (**B**) for Alzheimer’s disease (AD) patients, and (**C**) frontotemporal dementia (FTD) patients. Note that the scale is different for each group to facilitate the representation of the differences

The mean CAI for the follow-up visits was 0.43 ± 0.31 for AD patients, 0.82 ± 0.77 for FTD patients, and 0.16 ± 0.12 for CTR. At the longitudinal level (Fig. 3) with the participants with two visits, we found that CAI significantly increased in FTD, indicating a significantly more asymmetric brain over time (*p*-value = 0.019 between baseline and follow-up, measured as the interaction with age). We did not find statistically significant differences between visits in AD patients and CTR. The same analysis was performed with all the participants, leading to the same results (see Supplementary Material).

### Cluster group

**Fig. 4:**
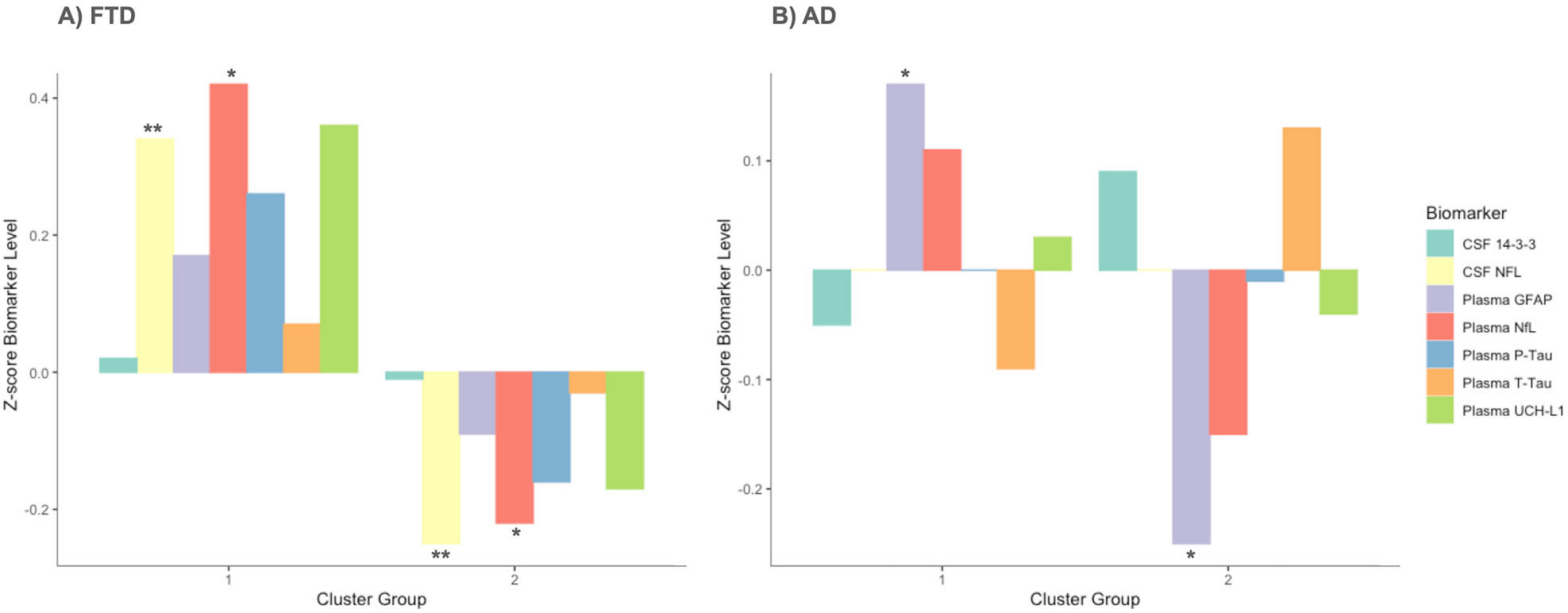
Mean *z*-scores of each biomarker level within each group. **A** represents the frontotemporal dementia patients (FTD) participants, and **B** represents the Alzheimer’s disease (AD) participants. Cluster 1, in both cases, is the most asymmetric. The symbol represents *p < 0.05 and **p < 0.01

The cluster analysis was used to identify subgroups based on their CAI. Then, we compared biomarker values between clusters. This method could divide the FTD group into Cluster 1 (*N* = 41) and Cluster 2 (*N* = 58) (see Supplementary Material Fig. 2 with the distribution of CAI according to each cluster). Cluster 1 presented a more asymmetric brain with 62.5% svPPA, 35.2% bvFTD, and 33.3% nfvPPA of the total cohort participants. In contrast, Cluster 2 showed a less asymmetric brain and grouped as 37.5% svPPA, 64.8% bvFTD, and 66.7% nfvPPA of the total cohort participants. CSF and plasma levels of NfL were increased in Cluster 1 compared to Cluster 2 (*p*-value = 0.0059 and *p*-value = 0.042, respectively) (Fig. 4A).

Within AD participants, the cluster analysis also yielded two groups (see Supplementary Material Fig. 2 with the distribution of CAI according to each cluster), presenting differences in plasma GFAP levels (*p*-value = 0.035) (Fig. 4B). Then, we tried to find an explanation based on the clinical profile to explain the clusters. However, we did not find any difference between clusters at a clinical level. The distribution of the participants in the two clusters was not grouped due to sex, age of onset (early-onset AD vs. late-onset AD), age of disease duration, AD phenotypes (amnesic vs. non-amnesic), MMSE, APOE status, or the combination of these variables.

### Relation between CAI, fluid biomarkers, and cognitive measures

**Fig. 5:**
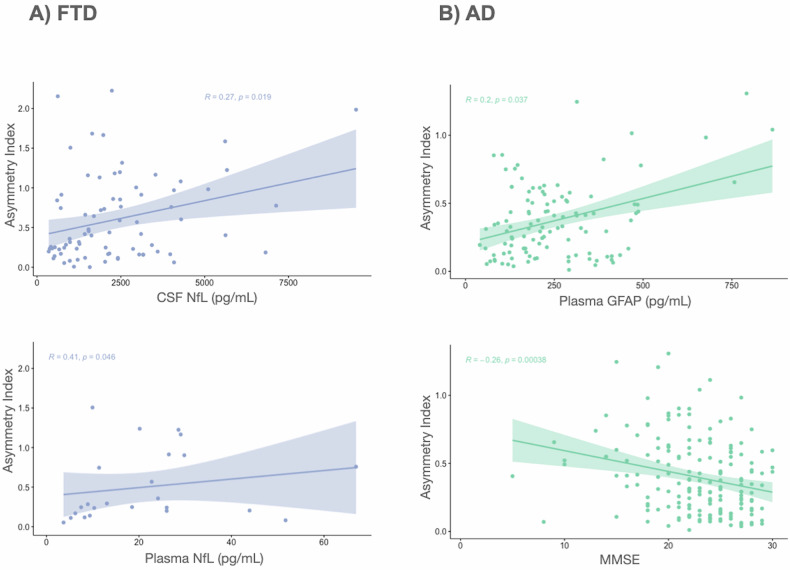
Correlation plots between the Cortical Asymmetric Index and biochemical biomarkers levels, as well as Mini-Mental State Examination (MMSE) scores. We only show the ones that reached statistical significance (*p*-value < 0.05). **A** Frontotemporal dementia patients (FTD) participants and (**B**) Alzheimer’s disease (AD) patients

We assessed whether CAI values correlated with the levels of the fluid biomarkers that emerged in the cluster analysis, in addition to MMSE and age (Fig. 5). We found a weak positive correlation in AD for plasma-GFAP (*r* = 0.20, *p*-value < 0.05) and a weak negative correlation with MMSE (*r* = −0.26, *p*-value < 0.001). For FTD patients, we found a significant positive correlation of CAI values with NfL levels; both CSF-NfL (*r* = 0.27, *p*-value < 0.05) and plasma-NfL (*r* = 0.41, *p*-value < 0.05). We found no correlation between CAI and fluid biomarkers in the different FTD clinical phenotypes. CTR did not present any correlation between the CAI and age either. Age did not correlate with CAI in any of the groups.

## Discussion

In this study, we propose a methodology to estimate structural brain asymmetries, namely the CAI, for neurodegenerative diseases, especially FTD and AD. We used an information theory method to calculate each individual’s CAI. CAI mean values differentiate CTR from dementia participants, FTD from AD, and FTD phenotypes. We studied the brain asymmetries over time and found an increase in asymmetry for FTD. Cluster analysis using the CAI values for both FTD and AD enabled us to identify disease subgroups and study the relationship between brain asymmetry and the levels of fluid biomarkers.

Our first finding, increased asymmetry for FTD, aligns with what has been described in the literature using a mix of non-standardised measures and visual inspection. The svPPA is FTD’s most asymmetrical clinical phenotype, usually showing greater left than right atrophy of the temporal lobes [6, 8, 10, 12, 32–36]. Also, in agreement with our findings, the FTD brain asymmetry has been reported to increase over time [10, 36]. In contrast, AD is commonly described as symmetric dementia: the atrophy presented in one hemisphere of the brain, usually, is similar in the other. However, some studies reported asymmetric patterns in AD [34, 37–40]. In our study, AD patients present lower CAI when compared with FTD patients, meaning less asymmetry. However, if compared to CTR, they present an asymmetrical brain.

Previous works studying brain asymmetries in FTD and AD usually report these asymmetries at the visual level. Still, further studies are needed to present a methodology to evaluate and quantify systematically. Although some studies have been done to quantify asymmetries [32, 35, 38, 41, 42], we chose a more complex methodology based on information theory to estimate an asymmetric index. For this reason, we proposed estimating the asymmetric index with a variation of the JSD measure. This novel measure based on entropy has recently been used to study similarities in different biological and clinical areas. For example, it has been used to help in the detection of ischaemic stroke, to study long-term surgical outcomes in the brain for epilepsy patients, to study cancer cells to find link cells with near-identical gene expression, or to study the individual metabolic network in patients with type 2 diabetes [22, 43–45]. We demonstrated that the CAI defined here could be of help for the differentiation of the clinical expressions or dementias, for studying the progression of the disease, or for identifying subgroups.

Other studies have explored quantification strategies for brain symmetries to differentiate between different dementias successfully [32, 46]. However, there is currently no standard and accurate methodology for this, and the CAI presented here showed promising results. Also, it helped identify FTD clinical phenotypes. In this sense, svPPA presented differences compared to bvFTD. We found that the svPPA is the most asymmetric clinical expression of FTD, which is in accordance with previous literature using visual scales [8, 9]. We replicated the previous results in this study and quantified these differences using the CAI.

Our cluster analysis yielded FTD and AD participants into two subgroups according to their asymmetry indexes. As cluster analysis is an unsupervised statistical approach, we aimed to find an explanation of the identified subgroups using clinical and biomarker data. Among the two subgroups for FTD patients, the more asymmetric cluster was enriched with svPPA participants, and the other cluster was enriched with nfvPPA and bvFTD. The cluster analysis was in accordance with the differences studied between FTD clinical phenotypes using ANOVAs, where we found a significant difference between svPPA and bvFTD. Overall, we observed that svPPA presented the highest CAI compared to nfvPPA and bvFTD, although only the comparison with bvFTD was significant. AD participants were also subdivided into two subgroups; however, we could not find an explanation based on the clinical profile of the subjects. We then analysed the association between clusters and the fluid biomarkers to study further implications of cortical brain asymmetry. We found that in FTD Cluster 1 (svPPA), CAI values were associated with higher levels of NfL in both CSF and plasma compared to Cluster 2 (bvFTD and nfvPPA). Furthermore, higher CAI predicted higher NfL (CSF and plasma) in FTD patients. This suggests that NfL (CSF and plasma) is directly associated with brain asymmetry. Previous studies reported that NfL levels in CSF and plasma were associated with brain atrophy [47–49]. However, the association between NfL and brain asymmetries has not been investigated. When studying AD, we found that different CAI groups were associated with plasma GFAP levels. This biomarker has been previously associated with brain atrophy due to ageing or disease severity [50, 51]. However, again, its association with brain asymmetry has not been studied before. Then, we examined the correlation between GFAP levels and CAI in AD patients and obtained a positive correlation. Overall, these associations between brain asymmetries and fluid biomarkers suggest that both contribute to defining AD subgroups.

Finally, FTD participants presented higher levels of brain asymmetry over time, suggesting that the CAI could indicate FTD progression. Previous studies have shown that FTD’s different genetic or clinical expressions behave differently in becoming more asymmetric over follow-up visits [52–54]. Regarding AD, no significant differences in CAI over time were found for AD patients. Even though there is no statistical difference, AD patients’ CAI should increase at some stage of the disease, as AD patients present increased CAI respecting CTR.

Although CAI is a good methodology to measure cortical brain asymmetry and help differentiate between dementias or subtypes, it presents some limitations. The sample size is a limitation to study a more direct evaluation of the sensitivity and specificity of CAI, with a larger cohort that could be studied. Also, the CAI is a more mathematically complex method than other asymmetry indexes, so it can be more challenging to use in clinical practice.

In conclusion, the proposed CAI measure might identify differences between AD and FTD and between FTD phenotypes and track progression in FTD. Cortical asymmetry drives subgrouping inside FTD and AD, associated with various fluid biomarker levels. Overall, this highlights the potential relevance of quantifying cortical asymmetry. Further studies investigating the predictive value of the CAI towards clinical progression might be of interest.

## Supplementary information

ELECTRONIC SUPPLEMENTARY MATERIAL

## Abbreviations

AD: Alzheimer’s disease
bvFTD: Behavioral variant frontotemporal dementia
CAI: Cortical Asymmetry Index
CSF: Cerebrospinal fluid
CTh: Cortical thickness
CTR: Healthy controls
FTD: Frontotemporal dementia
GFAP: Glial fibrillary acidic protein
HCB: Hospital Clínic de Barcelona
MMSE: Mini-Mental State Examination
MRI: Magnetic resonance imaging
NfL: Neurofilament light chain
nfvPPA: Nonfluent variant primary progressive aphasia
p-tau: Phospho tau
svPPA: Semantic variant primary progressive aphasia
t-tau: Total tau––
UCH-L1: Ubiquitin C-terminal hydrolase L1
