# Supplementary material for: The Cortical Asymmetry Index for subtyping dementia patients
Source: Eur Radiol. 2025 Feb 11;35(8):4713–21. doi: 10.1007/s00330-025-11400-y (PMC12226600; doi:10.1007/s00330-025-11400-y)
Supplement: Supplementary file 1 — ELECTRONIC SUPPLEMENTARY MATERIAL [file 330_2025_11400_MOESM1_ESM.pdf]

# The Cortical Asymmetry Index for subtyping dementia patients

## ELECTRONIC SUPPLEMENTARY MATERIAL

**Sex effect on CAI:**

At the cross-sectional level, adding sex to the analysis of group differences does not affect the analysis and leads to the same results. The participant's sex does not affect CAI.

**Brain size effect on CAI group differences:**

We study the effect of the total brain volume (as the intracranial volume) or mean cortical thickness on CAI group differences. Adding these two variables or one of them maintains the group's differences (AD, FTD, CTR) to an adjusted p-vale<0.0001. Thus, these two variables do not affect CAI group differences.

**Longitudinal changes in CAI**

Using all the study participants at the longitudinal level (Figure 1 Supplementary Material), we found that CAI significantly increased in FTD, indicating a significantly more asymmetric brain over time (p-value=0.012 between baseline and follow-up, measured as the interaction with age). We did not find statistically significant differences between visits in AD patients and CTR.

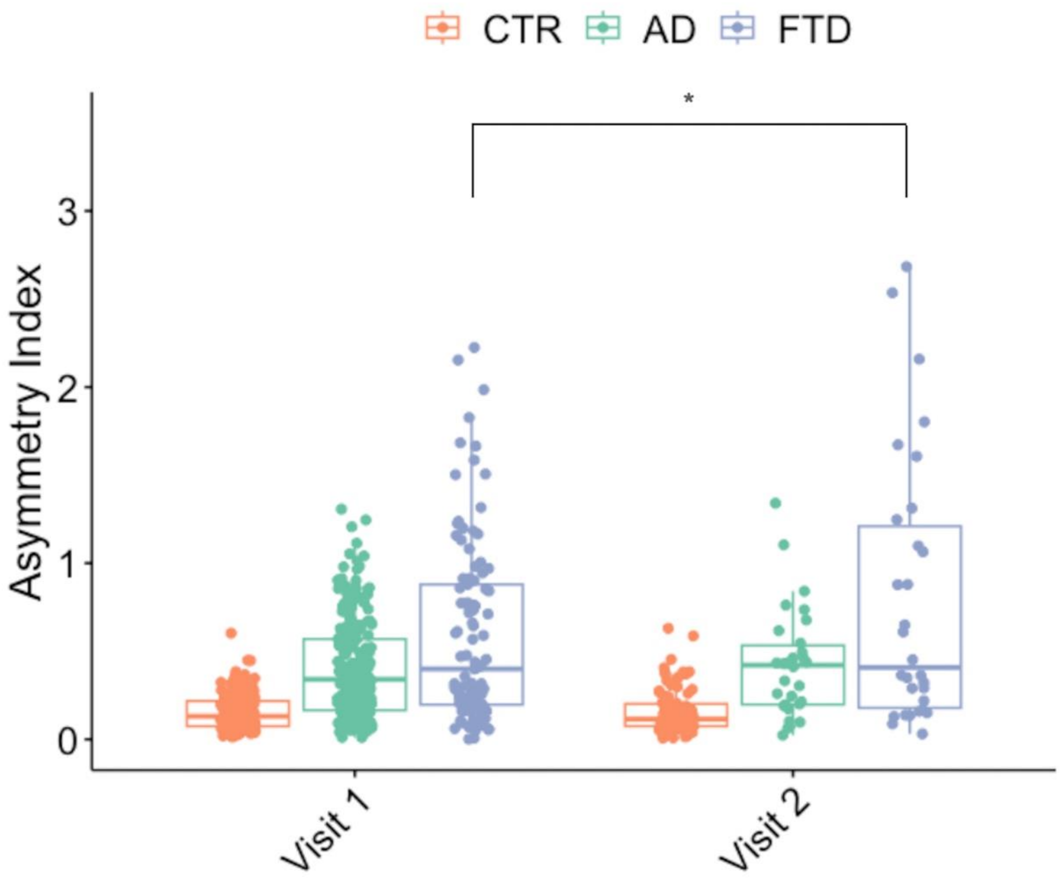


*Figure 1: Boxplot for Alzheimer's Disease (AD), frontotemporal dementia (FTD), and healthy controls (CTR) for two different visits. The symbol represents * p<0.05.*

**Cluster Group**

The agglomerative coefficient (AC) in the AD cluster was 0.9990, and in FTD, it was 0.997. Thus, our cluster analysis presents a strong structure in both cases.

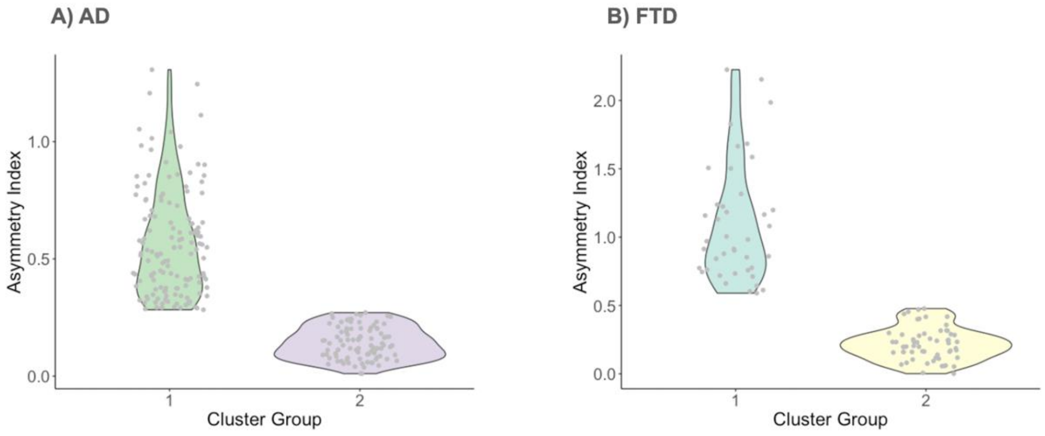


*Figure 2: Violin plots represent the distribution of CAI according to each cluster. A) Alzheimer's Disease (AD) and B) frontotemporal dementia (FTD),*
